# Influencing Factors, Construction and Verification of a Nomogram Model for Adolescent Depression With Nonsuicidal Self-Injury Behaviour

**DOI:** 10.62641/aep.v54i1.2007

**Published:** 2026-02-15

**Authors:** Fanfan Lu, Meihua Li, Zhengmao Cai, Xinxin Huang

**Affiliations:** ^1^Mental Health Center For Children And Adolescents, The Affiliated Kangning Hospital of Wenzhou Medical University, Zhejiang Provincial Clinical Research Center for Mental Health, 325000 Wenzhou, Zhejiang, China

**Keywords:** adolescent depression, nonsuicidal self-injury, influencing factors, nomogram model, emotion regulation, neuroimaging

## Abstract

**Background:**

Adolescent depression with nonsuicidal self-injury (NSSI) is a serious public health issue. NSSI involves intentional self-harm without suicidal intent and is common amongst depressed teens, leading to considerable psychological and physical risks. Early detection and intervention are essential to reduce these risks. To explore the influencing factors of adolescent depression with NSSI behaviour and construct a nomogram prediction model and verify its clinical application value.

**Methods:**

From January 2023 to April 2025, 136 cases of adolescent depression admitted to our hospital were selected. Patients were randomly divided into training (n = 95) and verification (n = 41) sets in a 7:3 ratio. Multivariate logistic regression was used to analyse the risk factors of NSSI behaviour in the training set, and a nomogram prediction model was constructed. Receiver operating characteristic (ROC) and calibration curves were drawn to evaluate the prediction efficiency of the nomogram model, and verification was conducted on the basis of the verification set. Decision curve analysis was applied to assess the clinical application value of the nomogram model for the prediction of NSSI behaviour.

**Results:**

The training and verification sets included 38 (40.00%) and 15 (36.59%) of cases of NSSI behaviour, respectively. No statistically significant differences in the incidence and clinical characteristics of NSSI behaviour were found between the training and verification sets (*p* > 0.05). Multivariate logistic regression analysis on the training set indicated that tense parental relationship, long depression duration, co-occurring physical diseases, high depression severity, high anxiety levels, childhood trauma, Electroencephalogram (EEG) frontal α power, functional Magnetic Resonance Imaging (fMRI) dorsolateral prefrontal cortex activation and negative life events were factors associated with NSSI behaviour (*p* < 0.05). The nomogram model showed good calibration and fit between prediction and reality on the training and verification sets with C-index values of 0.936 and 0.923, respectively. average absolute errors between predicted and actual values of 0.092 and 0.105, respectively. and Hosmer–Lemeshow test *p* values of 0.452 and 0.523, respectively. ROC curves indicated that the areas under the curve of the nomogram model for predicting the NSSI behaviour of patients with adolescent depression in the training and verification sets were 0.941 (95% CI: 0.887–0.995) and 0.928 (95% CI: 0.834–1.000), respectively, with the sensitivity of 0.929 and 0.846, respectively, and specificity of 1.000 and 0.667, respectively.

**Conclusion:**

The nomogram prediction model based on risk factors for depression with NSSI behaviour is beneficial for the early prediction of such behaviour in adolescents with depression, guiding appropriate clinical decisions and minimising the risk of NSSI behaviour, thereby safeguarding adolescent mental health.

## Introduction

Adolescence is a critical stage of human development. However, the incidence of adolescent depression, which poses a serious threat to the physical and mental health of adolescents, is increasing annually [1]. Adolescent depression with nonsuicidal self-injury (NSSI) behaviour has aroused widespread concern. NSSI behaviour refers to the intentional and repetitive injury, such as cutting, biting, scratching or hitting, to one’s body tissue without the intent to commit suicide [2]. This behaviour, which is common in patients with adolescent depression, not only exacerbates their suffering and healthcare burden but also elevates the risk of suicide, thereby dealing a heavy blow to families and society. This kind of behaviour is common in patients with adolescent depression, and its occurrence is closely related to risk factors under various theoretical frameworks. For example, family environment theory points out that tension between parents can destroy the emotional support system of adolescents, and previous studies have shown that family conflicts are significantly related to NSSI [3]. Traumatic stress theory holds that childhood trauma affects an individual’s ability to cope with stress by changing the development of the emotional regulation centre of the brain [4]. Life event theory states that negative life events can aggravate depressive symptoms and reduce psychological resilience. this effect then becomes the direct cause of NSSI [5]. According to emotional disorder theory, the degree of depression and anxiety directly reflects the disorder of an individual’s emotional regulation, and the comorbid state of high depression and anxiety is the core predictor of NSSI [6]. At present, the research on forecasting models has developed from single-factor analysis to multidimensional integration. For example, Șipoș *et al*. [5] built a machine learning–based risk model of adolescent NSSI. However, they only included psychosocial factors and did not combine clinical indicators. These models generally have the problems of high sample heterogeneity and insufficient clinical operability. In particular, they lack specific prediction tools for adolescent depression groups. New evidence shows that NSSI affects 12%–20% of teenagers around the world, and the prevalence rate of patients with depression is as high as 40%. NSSI behaviour not only leads to direct physical injuries, such as infections and scarring, but also causes long-term psychological problems, such as enhanced suicidal ideation and emotional adjustment disorder [7].

Although NSSI in depressed adolescents is a major public health challenge, doubling the risk of suicide attempts and imposing heavy burdens on families and society, the specific pathogenic pathway between adolescent depression and NSSI remains unclear. Existing studies mostly focus on isolated risk factors, such as family conflict and trauma, and lack a comprehensive predictive framework [8, 9]. This study aims to explore the influencing factors of NSSI in adolescents with depression and construct a validated nomogram prediction model. As a visual tool, this model integrates multidimensional risk factors (including psychosocial characteristics, clinical indicators and neuroimaging data) to provide clinicians with intuitive risk prediction results, filling the gap in NSSI prediction tools for adolescents with depression. It offers a quantitative basis for early intervention, ultimately contributing to the enhanced protection and promotion of adolescent mental health and reduced adverse outcomes.

## Materials and Methods

### Materials

A total of 136 patients with adolescent depression were selected from January 2023 to April 2025. All patients met the diagnostic criteria for major depressive disorder as defined by the Diagnostic and Statistical Manual of Mental Disorders, Fifth Edition (DSM-5) and provided voluntary informed consent. Subsequently, using a screening for non-suicidal self-injury (NSSI) behavior (defined as ≥5 episodes of self-injury in the past year), the patients were divided into two groups: the NSSI group (n = 53, with definite self-injury behavior) and the control group (n = 83, without self-injury behavior) [5]. Sample size estimation was based on the principle of the number of variables required by the logistic regression model in previous literature (each independent variable needs at least 10–15 samples). This study hypothesized seven potential risk factors (including parental relationships, prolonged depression, physical comorbidities, severe depressive and anxiety symptoms, childhood trauma, and negative life events) and the expected sample size needed 70–105 samples. In consideration of a shedding rate of 20%, 136 cases were finally included. Inclusion criteria included the following: (1) aged between 12–18 years. (2) met the diagnostic criteria for depression in the DSM-5 [10]. and (3) patients and their families were informed and agreed to cooperate with the investigation. Exclusion criteria encompassed the following: (1) co-occurring other mental disorders or physical diseases. (2) history of suicidal behaviour. (3) inability to complete the questionnaires because of intellectual disabilities (such as mild or severe intellectual disability and autism spectrum disorder). and (4) participation in a study and evaluation of the influence of or suffering from serious physical diseases (such as malignant tumours and organ failure). This research strictly followed the ethical guidelines of the Helsinki Declaration. All data were anonymised (identifiers, such as name and ID number, were deleted and replaced with research IDs), and only researchers were authorised to access the data. The patient and their legal guardian signed the informed consent form, which was approved by the affiliated kangning hospital of wenzhou medical university ethics committee (approval number: YJ-2025-11-02).

Both groups excluded those who had attempted suicide. Patients were randomly divided into the training (n = 95) and validation (n = 41) sets in a 7:3 ratio. The sample size met the requirement of 10–15 samples per independent variable (seven factors), ensuring statistical power. Internal validation was performed to assess model stability.

### Methods

#### Data Collection

Demographic data included gender, age, ethnicity, family type, parental relationship, place of residence, only-child status and history of being left behind.

Clinical features comprised age of onset, duration of illness, history of trauma, presence of co-occurring physical illness, severity of depression and level of anxiety. The severity of depression was assessed by employing the Hamilton Depression Rating Scale (HAMD) [11], and the level of anxiety was assessed by using the Hamilton Anxiety Scale (HAMA) [12].

Psychosocial factors encompassed childhood trauma, negative life events, family relationships and social support. Childhood trauma was assessed by using the Childhood Trauma Questionnaire (CTQ) [4], and negative life events were assessed with the Adolescent Life Events Scale (ASLEC) [13].

Neurobiological measures were acquired as follows: 64-channel Electroencephalogram (EEG) (Brain Products GmbH, Gilching, Germany): Resting-state brain activity was recorded at a sampling rate of 1000 Hz with Ag/AgCl electrodes placed in accordance with the International 10–20 System. EEG frontal α power was measured bilaterally, and the average value was used for analysis (unit: µV^2^). 3T frontal α power, functional Magnetic Resonance Imaging (fMRI) (model: MAGNETOM Prisma, Siemens Healthineers, Erlangen, Germany): During functional scanning, participants completed an emotional face processing task (presenting angry/happy neutral faces). Blood-oxygen-level-dependent (BOLD) signals were acquired by using a T2*-weighted echo planar imaging sequence (TR/TE = 2000/30 ms, 64 × 64 matrix, 33 axial slices). Amygdala and dorsolateral prefrontal cortex (DLPFC) (Unit: % BOLD signal change) activation was analysed by using SPM12 (Version 12. Wellcome Trust Centre for Neuroimaging, London, UK).

#### HAMD

The HAMD is a classic tool for evaluating the severity of depressive symptoms. It is widely used in psychiatric clinical and scientific research fields. Although it was originally developed for adults, previous studies have verified its applicability in adolescents and proved its good internal consistency (Cronbach’s α = 0.82–0.89) and convergence effectiveness with child-specific scales, such as the Child Depression Scale [11]. Its items are diverse, covering a wide range of dimensions, such as low mood (frequency of despair), loss of interest (diminished enthusiasm), sleep disorders (difficulty falling asleep and sleep quality), appetite changes (fluctuations in appetite and weight) and suicidal ideation (presence and intensity). On the basis of a standardised process, symptoms are scored on a 0–4 scale, with total scores ranging from 0 to 60. Mild depression (8–17 points) has slight symptoms and slightly disturbed functioning. moderate depression (18–24 points) has considerable functional impairment, making daily tasks difficult. and severe depression (≥25 points) has severely impaired functioning, almost losing self-care ability, with a dramatically increased risk of suicide.

#### HAMA

HAMA is a key tool for measuring anxiety levels. It precisely dissects somatic and psychic anxiety [12]. Cross-cultural validation studies amongst Asian teenagers reported acceptable reliability (intraclass correlation coefficient = 0.78–0.85) and structural validity, although the wording of the project (such as ‘work difficulty’) was suitable for the background of teenagers. Somatic anxiety includes muscle tension (location, frequency and intensity), such as shoulder pain episodes. cardiovascular symptoms (palpitation frequency and severity). respiratory symptoms (shortness of breath). and gastrointestinal symptoms (frequency of nausea and abdominal pain). Psychic anxiety focuses on anxious mood (duration and intensity of worry and fear), tension (restlessness and irritability) and panic attacks (presence and characteristics). Scores are aggregated on a 0–4 scale, with a total score ranging from 0 to 56. Typically, mild anxiety (7–13 points) involves occasional anxiety experiences without remarkable life disruption. moderate anxiety (14–20 points) involves frequent anxiety symptoms, with some functional impairment. and severe anxiety (21 points and above) is generalised, with near-paralysis in various aspects of life, urgently needing intervention for relief.

#### CTQ

CTQ is designed to assess the severity of childhood trauma across five dimensions: emotional abuse (frequent verbal humiliation), physical abuse (details of physical punishment and beating), sexual abuse (frequency and nature of contact), emotional neglect (indifference to emotional needs) and physical neglect (degree of inadequate care). It consists of 28 items scored on a five-point Likert scale (total score range: 25–125) [4]. High scores indicate severe experiences of childhood trauma. In this study, CTQ scores were evaluated on the basis of the total score: a score below 40 indicates mild trauma, that between 40 and 79 specifies moderate trauma and that of 80 or above reflects severe trauma.

#### ASLEC

ASLEC evaluates negative life events on a 0–6 scale (total score range: 0–78) [13]. It systematically evaluates the impact of negative life events across multiple domains, including academics (e.g., exam failure and heavy academic pressure), interpersonal relationships (e.g., conflicts, bullying and strained relationships), family (e.g., crises or changes) and health (e.g., serious or chronic illnesses). Each event is rated on a 0–6 scale to assess its occurrence and the degree of psychological and life impact on the adolescent. High total scores reflect a strongly negative influence. Total scores below 35 indicate mild impact, typically presenting as temporary emotional fluctuations that can be self-regulated. scores between 35 and 65 suggest moderate impact, with noticeable emotional and behavioural changes and partial functional impairment requiring long recovery. and scores above 65 indicate severe impact, potentially leading to stress-related disorders and requiring professional intervention to restore psychological balance. The scale is useful for identifying high-risk adolescents and providing targeted guidance for psychological support.

#### Statistical Analysis

SPSS 26.0 (IBM, Armonk, New York, USA) software was used for data analysis. The Kolmogorov–Smirnov test was conducted to test normality. Measurement data conforming to the normal distribution were expressed as means with standard deviations ($\bar{x}$ ± s), and the independent samples *t*-test was employed for comparison between groups. Data with nonnormal distribution were analysed after logarithmic transformation or expressed as medians (interquartile interval), and the Mann–Whitney U test was employed for comparison between groups. Categorical variables were presented as frequencies and percentages, with group differences assessed through the χ^2^ test. Univariate and multivariate logistic regression analyses were conducted to identify factors influencing depression amongst adolescents with NSSI behaviour. R software (version 3.5.2, R Foundation, Vienna, Austria) was employed to standardise continuous variables (depression severity, anxiety, trauma, life events, EEG α wave power and functional Magnetic Resonance Imaging-Dorsolateral Prefrontal Cortex (fMRI-DLPFC) activation) to z-scores, and a nomogram model based on regression coefficient allocation point values was constructed by utilising the lrm function. Model performance was evaluated by utilising the C-index, calibration curve (Hosmer–Lemeshow test) and decision curve analysis (DCA) with the help of Receiver Operating Characteristic (ROC) and the decision curve package. Two-sided *p* < 0.05 was considered statistically significant.

## Results

### Comparison of the Incidence and Clinical Characteristics of NSSI Behaviour Between the Training and Verification Sets

In the training set of 95 patients, 38 (40.00%) exhibited NSSI behaviour, whereas in the validation set of 41 patients, 15 (36.59%) showed this behaviour. No significant differences were found in incidence and clinical characteristics between the training and verification sets (*p* > 0.05), as shown in Table 1.

**Table 1.** **Comparison of clinical characteristics between the training and verification sets**.

Index		Training set (n = 95)	Verification set (n = 41)	*t/χ^2^*	*p*
Age ($\bar{x}$ ± s, years)		15.32 ± 1.80	15.03 ± 1.65	0.883	0.378
Gender [n (%)]	Male	45 (47.37)	18 (43.90)	0.138	0.709
	Female	50 (52.63)	23 (56.10)		
Family type [n (%)]	Nuclear family	60 (63.16)	21 (51.22)	2.107	0.550
	Single parent family	15 (15.79)	7 (17.07)		
	Reconstituted family	10 (10.53)	7 (17.07)		
	Other	10 (10.53)	6 (14.63)		
Parental relationship [n (%)]	Harmonious	40 (42.11)	18 (43.90)	0.116	0.943
	Average	40 (42.11)	16 (39.02)		
	Tense	15 (15.79)	7 (17.07)		
Depression duration [n (%)]	≤3 years	65 (68.42)	31 (75.61)	0.712	0.398
	>3 years	30 (31.58)	10 (24.39)		
Residence [n (%)]	Rural	30 (31.58)	12 (29.27)	0.071	0.789
	Urban	65 (68.42)	29 (70.73)		
Only child [n (%)]	Yes	51 (53.68)	19 (46.34)	0.618	0.431
	No	44 (46.32)	22 (53.66)		
Left behind experience [n (%)]	Yes	20 (21.05)	9 (21.95)	0.013	0.906
	No	75 (78.95)	32 (78.05)		
History of trauma [n (%)]	Yes	16 (16.84)	6 (14.63)	0.103	0.748
	No	79 (83.58)	35 (85.37)		
Co-occurring physical diseases [n (%)]	Yes	27 (28.42)	12 (29.27)	0.010	0.919
	No	68 (71.58)	29 (70.73)		
Severity of depression ($\bar{x}$ ± s, points)		22.36 ± 3.75	23.24 ± 3.46	1.284	0.201
Anxiety level ($\bar{x}$ ± s, points)		18.61 ± 2.89	19.34 ± 3.07	1.326	0.186
Childhood trauma ($\bar{x}$ ± s, points)		68.95 ± 9.03	69.03 ± 9.12	0.047	0.962
Negative life events ($\bar{x}$ ± s, points)		55.68 ± 7.69	56.42 ± 7.54	0.518	0.605
EEG frontal α power (µV^2^)		12.36 ± 3.75	12.14 ± 3.46	0.321	0.748
fMRI-DLPFC activation (% BOLD signal change)		0.85 ± 0.18	0.87 ± 0.15	0.623	0.533
Incidence of NSSI [n (%)]	Yes	38 (40.00)	15 (36.59)	0.140	0.707
	No	57 (60.00)	26 (63.41)		

Note: EEG, Electroencephalogram; fMRI-DLPFC, functional Magnetic Resonance Imaging-Dorsolateral Prefrontal Cortex; BOLD, Blood-oxygen-level-dependent; NSSI, nonsuicidal self-injury. The Kolmogorov–Smirnov test was used to test for normality. All measurement data conformed to a normal distribution (*p* > 0.05) and were expressed as mean ± standard deviation. Variables with *p* < 0.05 in univariate analysis are included as covariates in multivariate analysis.

### Associated Factor Analysis for NSSI in the Training Set

As shown in Table 2, the results of univariate analysis revealed that non-NSSI patients and patients with NSSI differed significantly in terms of parental relationship, depression duration, co-occurring physical diseases, depression severity, anxiety level, childhood trauma, EEG frontal α power, fMRI-DLPFC activation and negative life events (*p* < 0.05). Further multivariate logistic regression analysis was conducted with NSSI occurrence as the dependent variable (0 = no, 1 = yes) and factors with *p* < 0.05 from the univariate analysis as covariates (Table 3, Ref. [4, 13]). The results of multivariate logistic regression analysis are shown in Table 4.

**Table 2.** **Univariate analysis of NSSI in the training set**.

Index		NSSI group (n = 38)	Non-NSSI group (n = 57)	*t/χ^2^*	*p*
Age ($\bar{x}$ ± s, years)		15.03 ± 1.51	15.10 ± 1.42	0.229	0.819
Gender [n (%)]	Male	17 (44.74)	25 (43.86)	0.007	0.932
	Female	21 (55.26)	32 (56.14)		
Family type [n (%)]	Nuclear family	17 (44.74)	38 (66.67)	4.543	0.208
	Single-parent family	8 (21.05)	7 (12.28)		
	Reconstituted family	7 (18.42)	6 (10.53)		
	Other	6 (15.79)	6 (10.53)		
Parental relationship [n (%)]	Harmonious	10 (26.32)	30 (52.63)	12.210	0.002
	Average	12 (31.58)	20 (35.09)		
	Tense	16 (42.11)	7 (12.28)		
Depression duration [n (%)]	≤3 years	18 (43.37)	40 (70.18)	4.987	0.025
	>3 years	20 (52.63)	17 (29.82)		
Residence [n (%)]	Rural	14 (36.84)	23 (40.35)	0.118	0.731
	Urban	24 (63.16)	34 (59.65)		
Only child [n (%)]	Yes	19 (50.00)	32 (56.14)	0.345	0.556
	No	19 (50.00)	25 (43.86)		
Left behind experience [n (%)]	Yes	8 (21.05)	14 (24.56)	0.157	0.691
	No	30 (78.95)	43 (75.44)		
History of trauma [n (%)]	Yes	6 (15.79)	11 (19.30)	0.191	0.662
	No	32 (84.21)	46 (80.70)		
Co-occurring physical diseases [n (%)]	Yes	9 (23.68)	4 (7.02)	5.361	0.020
	No	29 (76.32)	53 (92.98)		
Severity of depression ($\bar{x}$ ± s, points)		24.64 ± 4.55	22.20 ± 4.18	2.690	0.008
Anxiety level ($\bar{x}$ ± s, points)		19.15 ± 3.87	17.54 ± 3.03	2.268	0.025
Childhood trauma ($\bar{x}$ ± s, points)		75.12 ± 10.10	65.68 ± 9.81	5.896	0.001
Negative life events ($\bar{x}$ ± s, points)		58.75 ± 8.82	50.36 ± 7.62	4.934	0.001
EEG frontal α power (µV^2^)		10.24 ± 4.55	12.80 ± 4.18	2.822	0.005
fMRI-DLPFC activation (% BOLD signal change)		0.71 ± 0.12	0.81 ± 0.13	3.786	0.001

Note: EEG, Electroencephalogram; fMRI-DLPFC, functional Magnetic Resonance Imaging-Dorsolateral Prefrontal Cortex; BOLD, Blood-oxygen-level-dependent; NSSI, nonsuicidal self-injury.

**Table 3.** **Variable description and measurement scale**.

Index	Meaning	Assignment/measurement scale
Parental relationship	State of parent interaction in the family	0 = harmonious, 1 = average, 2 = tense
Depression duration	Duration of depression	0 = ≤3 years, 1 = >3 years
Co-occurring physical diseases	Are there any other physical diseases?	0 = no, 1 = yes
Severity of depression	Severity of depressive symptoms	Continuous variable (HAMD score, ranging from 0 to 60)
Anxiety level	Severity of anxiety symptoms	Continuous variable (HAMA score, ranging from 0 to 56)
Childhood trauma	Severity of traumatic events experienced in childhood.	Continuous variable (CTQ score, ranging from 0 to 125 [4])
Negative life events	Influence degree of negative life events	Continuous variable (ASLEC score, ranging from 0 to 78 [13])
EEG frontal α power	Power of frontal lobe α band (8–12 Hz)	Continuous variable (unit: µV^2^)
fMRI-DLPFC activation	Power of frontal lobe α band (8–12 Hz)	Continuous variable (unit: % BOLD signal change)
NSSI occurrence	Is there any nonsuicidal self-injury behaviour?	None = 0, yes = 1

Note: NSSI, nonsuicidal self-injury; HAMD, Hamilton Depression Rating Scale; HAMA, Hamilton Anxiety Scale; CTQ, Childhood Trauma Questionnaire; ASLEC, Adolescent Life Events Scale; EEG, Electroencephalography; fMRI-DLPFC, functional Magnetic Resonance Imaging-Dorsolateral Prefrontal Cortex.

**Table 4.** **Multivariate logistic regression analysis for NSSI occurrence in the training set**.

Factor	B	Standard error	Wald	*p*	OR	95% CI	Collinear statistics	
							Tolerance	VIF
Parental relationship (average vs. harmonious), [n (%)]	0.523	0.132	4.012	0.132	1.682	1.305–2.168	0.287	3.749
Parental relationship (tense vs. harmonious), [n (%)]	0.662	0.271	5.950	0.015	1.939	1.139–3.300		
Depression duration (>3 year, [n (%)])	0.961	0.435	4.877	0.027	2.614	1.114–6.135	0.317	3.151
Severity of depression (HAMD, $\bar{x}$ ± s, points)	0.137	0.052	6.875	0.009	1.147	1.035–1.270	0.676	1.478
Anxiety level (HAMA, $\bar{x}$ ± s, points)	0.139	0.066	4.438	0.035	1.149	1.010–1.307	0.865	1.156
Childhood trauma (CTQ, $\bar{x}$ ± s, points)	0.097	0.025	14.513	0.001	1.102	1.048–1.158	0.911	1.098
Negative life events (ASLEC, $\bar{x}$ ± s, points)	0.132	0.033	15.565	0.001	1.141	1.069–1.218	0.895	1.117
Co-occurring physical diseases (yes, [n (%)])	1.414	0.644	4.824	0.028	4.112	1.164–14.523	0.728	1.373
EEG frontal α power (µV^2^)	−0.142	0.053	7.214	0.007	0.868	0.782–0.962	0.927	1.079
fMRI DLPFC activation (% BOLD signal change)	−4.261	1.731	6.058	0.014	0.014	0.001–0.420	0.961	1.041

Note: NSSI, nonsuicidal self-injury; VIF, Variance Inflation Factor; HAMD, Hamilton Depression Rating Scale; CTQ, Childhood Trauma Questionnaire; ASLEC, Adolescent Self-Rating Life Events Checklist; EEG, Electroencephalography; fMRI-DLPFC, functional Magnetic Resonance Imaging-Dorsolateral Prefrontal Cortex.

### Establishment of the NSSI Prediction Model

**Fig. 1.**
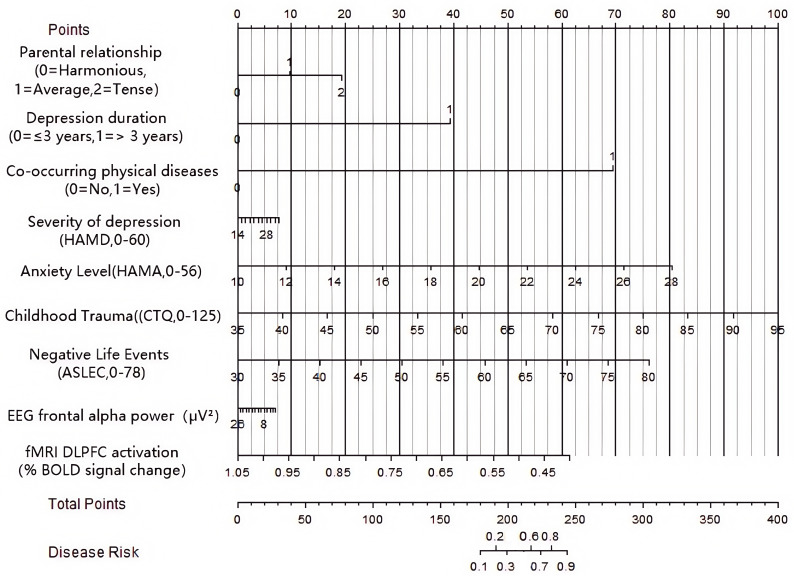
**Nomogram prediction model for NSSI occurrence in patients with adolescent depression**. NOTE: NSSI, nonsuicidal self-injury; VIF, Variance Inflation Factor; HAMD, Hamilton Depression Rating Scale; CTQ, Childhood Trauma Questionnaire; ASLEC, Adolescent Self-Rating Life Events Checklist; EEG, Electroencephalography; fMRI-DLPFC, functional Magnetic Resonance Imaging-Dorsolateral Prefrontal Cortex.

Continuous variables (depression severity, childhood trauma and negative life events) in the training set were tested for linear correlations on the basis of previous research evidence (such as those reported by Irniger C *et al*. [4]) and clinical professional cognition to judge the relationship between variables and the probability of NSSI. The results revealed an approximate linear correlation between continuous variables and NSSI risk (*p* < 0.05), and nonlinear transformation was not needed. On the basis of this result, the multivariate logistic regression analysis showed that tension with parents, long depression course, physical illness, high depression degree, high anxiety degree, childhood trauma, EEG frontal α power, fMRI-DLPFC activation and negative life events are the related factors of NSSI behaviour. For classified variables, the predictive contribution score was calculated in accordance with the β coefficient of logistic regression: the β value of each classification level was converted into the corresponding score (score = β value/minimum β value × 100). For example, the score of ‘nervousness’ (β = 0.662) in the parent–child relationship variable was (0.662/0.132) × 100 ≈ 50. The score of ‘average’ (β = 0.421) was (0.421/0.132) × 100 ≈ 32. Furthermore, to accurately estimate the predictive contribution of each category, we used effects coding (or contrast coding) instead of simple dummy coding (0–1). In this model, the ‘harmony’ category was designated as the reference group and assigned a value of 0 for comparison. This approach ensures that the coefficient for each other category represents its deviation from the overall mean, providing a more interpretable measure of its unique effect. Finally, the nomogram prediction model was constructed on the basis of the above score distribution. This method ensures that the length of the score axis of each variable in the nomogram is proportional to the β coefficient, and the positive/negative direction of the coefficient is reflected by the direction of the score axis (the right is a positive coefficient and the left is a negative coefficient). The model was constructed on the basis of the above standardised score distribution. The total score was used to predict the probability of NSSI, and a high total score is indicative of high prediction accuracy. Each related factor in the model was assigned points, and the total score of predicting NSSI was calculated and reflected by the probability of predicting NSSI. A high total score indicates the high accuracy of predicting NSSI in patients with adolescent depression (Fig. 1).

### Evaluation and Verification of the NSSI Prediction Model

**Fig. 2.**
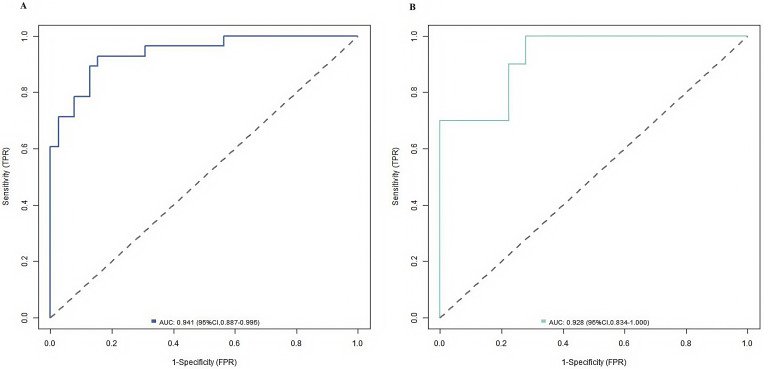
**Receiver operating characteristic(ROC) curve of the prediction model for NSSI in patients with adolescent depression ((A) is the training set, and (B) is the verification set)**.

**Fig. 3.**
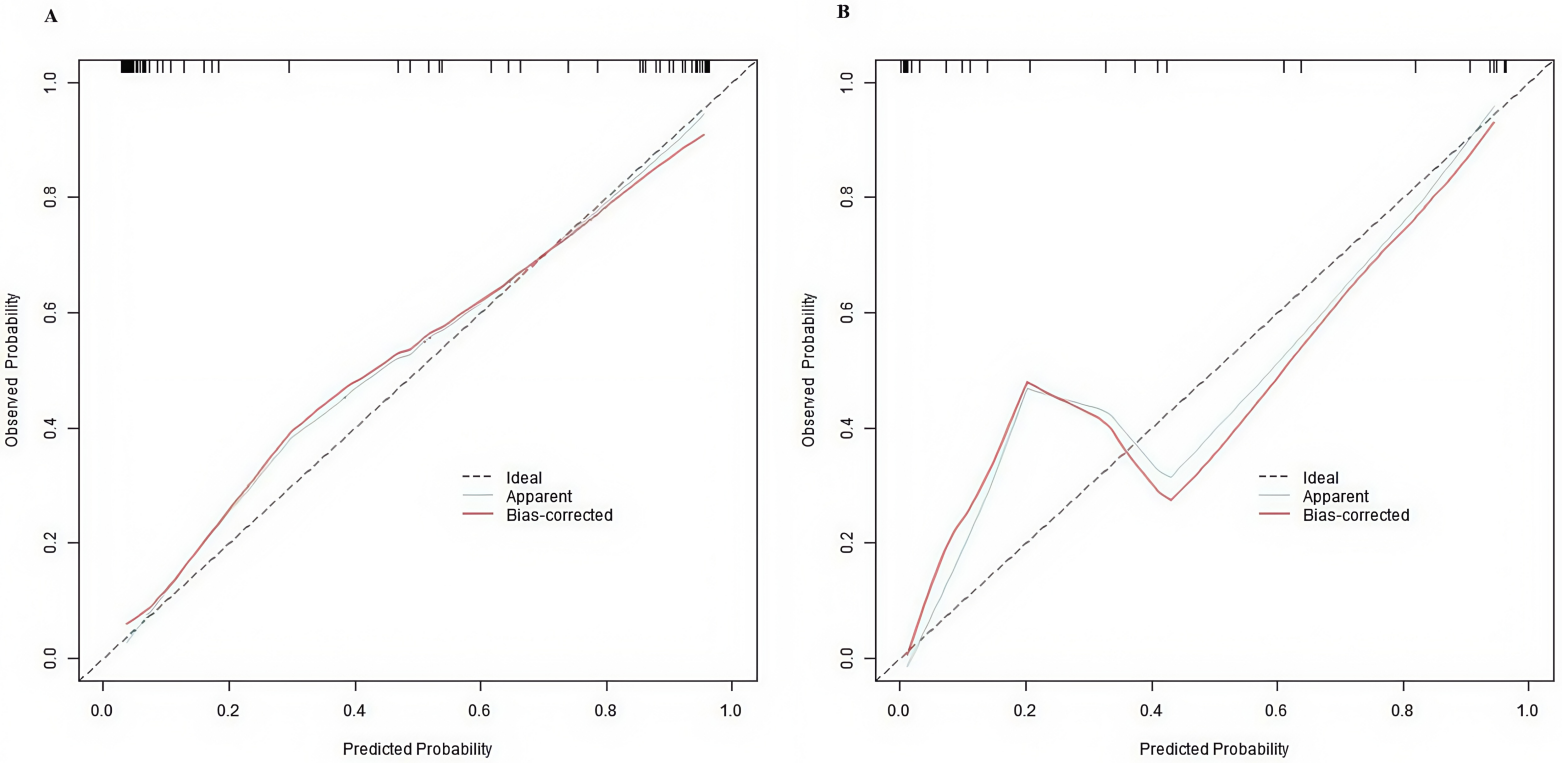
**Calibration curves of the prediction model for NSSI in adolescents with depression ((A) is the training set, and (B) is the verification set)**.

The nomogram model showed good calibration and fit on the training and verification sets (C-index values of 0.936 [95% CI: 0.887–0.995] and 0.923 [95% CI: 0.834–1.000]. average absolute errors between predicted and actual values of 0.092 and 0.105. and Hosmer–Lemeshow test *p* values of 0.452 and 0.523). ROC curves indicated that the area under the curve of the nomogram model in predicting NSSI behaviour in patients with adolescent depression in the training and verification sets were 0.941 (95% CI: 0.887–0.995) and 0.928 (95% CI: 0.834–1.000), respectively, with the sensitivity of 0.929 and 0.846, respectively, and specificity of 1.000 and 0.667, respectively. Fig. 2 shows ROC curves, and Fig. 3 presents calibration curves.

### DCA for the Nomogram Prediction Model

**Fig. 4.**
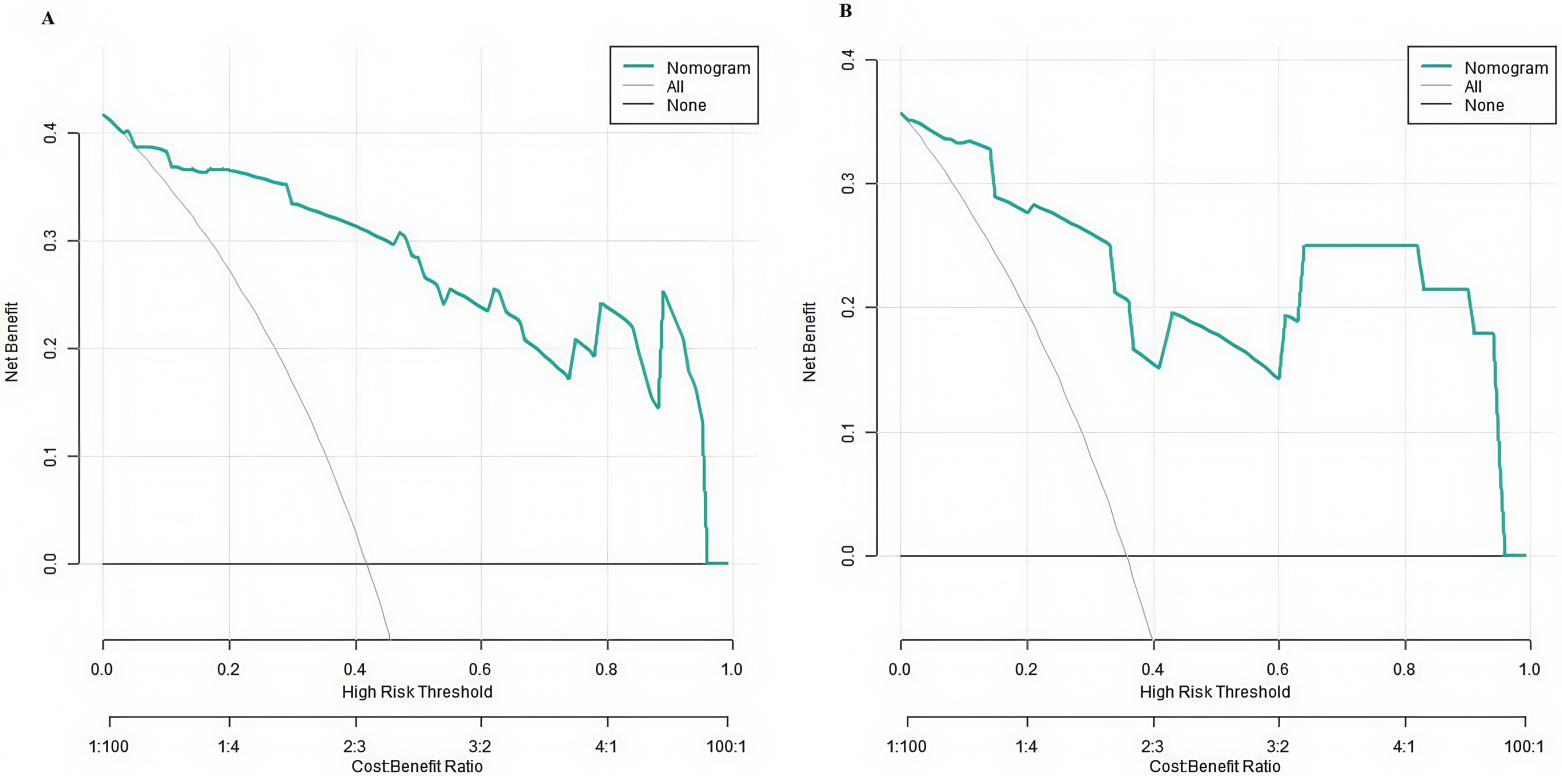
**Decision curve analysis (DCA) of the prediction model for NSSI in patients with adolescent depression ((A) is the training set, and (B) is the verification set)**.

The results of DCA showed that the clinical applicability of the nomogram model differed on different data sets. On the training set (Fig. 4A), the model showed significant net benefits in the threshold probability range of 0.15–0.85. However, on the verification set (Fig. 4B), the effective threshold range reduced to 0.20–0.75. This reduction may reflect the influence of sample fluctuation on the stability of the model. Notably, when the threshold probability exceeded 0.75, the net benefit of the ‘no intervention’ strategy was better than that predicted by the model. This finding suggested that clinicians should be cautious in applying the model results when evaluating high-risk patients. On the whole, the prediction model had the best clinical guidance value for patients in the middle-risk range (20%–75%). However, for high-risk patients (threshold >0.75), other clinical indicators for comprehensive evaluation should be combined to ensure the accuracy and safety of decision-making (Fig. 4).

## Discussion

In this study on adolescent depression with NSSI behaviour, multivariate logistic regression analysis identified tense parental relationships, long depression duration, co-occurring physical disease, high depression severity, high anxiety levels, childhood trauma and negative life events as the factors associated with NSSI behaviour. These factors reveal the complex causes of adolescents’ self-injury behaviour in the face of depression from different angles and are of great importance for deeply understanding the psychopathological mechanism of this special group and formulating accurate intervention strategies.

This study found that tense parental relationships (OR = 1.939) are a core risk factor for NSSI. This factor exhibits a more pronounced effect than family environment in ordinary adolescents observed by Liang *et al*. [3]. In the community samples of Liang *et al*. [3], the OR value of family conflict for NSSI was 1.52. The risk weight observed in depressed patients in the present study is higher than that observed by Liang *et al*. [3], suggesting that the pathological state of depression may exacerbate the negative impact of family tension through emotional dysregulation mechanisms. This study is the first to verify the independent predictive value of parental relationship tension in depression groups and to supplement the specific evidence of family factors in clinical samples. Long depression duration is also a crucial risk factor. With the prolongation of the course of depression, the psychological resilience of patients is constantly challenged, and many aspects, such as emotional regulation, cognitive function and social ability, are continuously damaged. Regarding the correlation between depression duration and NSSI, Nagy *et al*. [14] noted a correlation between chronic depression and self-injury but did not quantify the specific influence of depression duration. In the present study, logistic regression showed that the OR value for disease course > 3 years was 2.614, which is significantly higher than that (OR = 1.89) in Nagy *et al*. [14] meta-analysis. This difference is possibly due to the strict restriction to ‘depression with NSSI’ samples and the inclusion of neuroimaging indicators reflecting long-term brain function changes. Kang *et al*. [15] studied the family transmission of suicide and self-injury in low-income groups but did not discuss disease duration separately. The present study confirmed that the exhaustion of psychological resilience from long-term depression is an independent driver of NSSI, providing quantitative evidence for the cumulative effect of chronic depressive states, thus filling the gap in the research on the quantitative relationship between disease duration and self-injury. For adolescents with depression, co-occurring physical diseases are like adding insult to injury. Physical disease itself not only brings physical discomfort and limited function, but also increases the psychological burden, such as anxiety about the prognosis of the disease, pain during treatment and social isolation that may be caused by physical reasons, of patients. In contrast to those in the study of Fernando *et al*. [16], concomitant somatic diseases (OR = 4.112) show a very high risk weight in this study. Although this study focused on the influence of mental illness and somatic diseases on suicide, it did not analyse NSSI behaviour alone [16]. This study revealed the superposition effect of somatic diseases and depression in adolescents for the first time, and it reported an OR value that is considerably higher than that reported by Blessing *et al*. [17] for NSSI. This difference indicates that the impairment of physical function may promote self-injury through the two pathways of physical pain and psychological despair [17]. This discovery breaks through the limitation of previous studies focusing on psychosocial factors and brings the dimension of physical health into the NSSI prediction framework. High depression severity and anxiety levels are closely related and mutually influential, forming significant risk factors for NSSI. A high degree of depression means that patients are deeply immersed in negative emotions and cognitive states, such as depression, loss of interest, self-blame and self-guilt, and experience a strong sense of pain and helplessness. Meanwhile, a high degree of anxiety places them in a constant state of tension, anxiety and worry and fills them with fear for the future [6]. In terms of psychological mechanisms, NSSI in adolescent depression may be driven by a reciprocal pathway of emotion regulation difficulties and negative self-cognition. Emotion regulation dysfunction, such as impaired prefrontal–limbic network connectivity (e.g., reduced DLPFC control over the amygdala) [18] leads to failed emotional suppression, prompting individuals to use self-injury as an alternative regulatory strategy, as supported by the negative correlation between EEG frontal α power and NSSI (β = -0.142, *p* = 0.007) indicating disrupted frontal lobe emotional modulation. Meanwhile, a negative self-cognition schema, with childhood trauma–induced self-blame cognitions (CTQ score β = 0.097, *p* < 0.001) interacting with current depressive symptoms, forms a self-worth degradation cycle, where individuals with high self-blame tendencies may interpret negative life events (ASLEC score β = 0.132, *p* < 0.001) as self-validation of worthlessness, triggering self-injury as a form of emotional release or self-punishment [19, 20]. In summary, the pathway of emotion dysregulation → negative cognition → coping deficit underscores the complex psychological mechanisms underlying NSSI. these mechanisms are reflected in the model’s risk factors (e.g., anxiety, trauma and life events) [6, 18, 19]. Irniger C *et al*. [4] discussed the mediating effect of childhood trauma on NSSI through positive and negative coping styles but did not combine neuroimaging indicators. In this study, the total score of CTQ (OR = 1.102) and the α wave power of EEG frontal lobe were integrated synchronously. Trauma load could increase the risk of NSSI by regulating the emotional regulation network of the frontal lobe, thus revealing the pathological chain of trauma–neural mechanism–self–injury more deeply than the analysis of a single psychological scale. When adolescents later develop depression, their childhood traumatic experiences are easily reactivated, accounting for their lack of effective coping abilities when facing current psychological pressure. Traumatic emotions that were not properly handled in the past are intertwined with the pain caused by the current depression, greatly increasing the possibility of expressing inner pain through self-injury behaviour. Negative life events are another crucial factor [20]. These negative life events, whether they are major academic setbacks (such as exam failure and pressure to enter a high-ranking school), interpersonal conflicts (conflicts with classmates, teachers or family members), or family changes (such as the death of relatives and divorce of parents) exert a considerable psychological effect on adolescents, especially those who are already suffering from depression. Their already fragile psychological resilience can be overwhelmed by these additional stresses, prompting self-injury as a way to cope with inner pain and unresolved pressures [5]. This research demonstrates distinct innovations over recent studies. De Luca *et al*. [2] conducted a systematic review and Bayesian meta-analysis on NSSI amongst community adolescents, focusing on incidence trends, whereas the present study targeted depressed adolescents and innovatively integrated 64-channel EEG (frontal α-wave power) and 3T fMRI (DLPFC activation) into the NSSI risk prediction model [2]. By contrast, Guo *et al*. [21] only used single-mode fMRI to analyse prefrontal functional connectivity in patients with adolescent depression. This study, for the first time, constructed a multidimensional psychological–neural–clinical prediction model by integrating multimodal neuroimaging data (64-channel EEG and 3T fMRI), psychological scales (CTQ and ASLEC) and clinical risk factors (parental relationship tension and childhood trauma). It results show that the multimodal system significantly outperforms single-mode studies in terms of prediction efficacy, verifying the independent predictive value of seven factors (e.g., parental tension with OR = 1.939 and childhood trauma with OR = 1.102). This work provides a comprehensive tool for the risk stratification of adolescent depression with NSSI.

On the basis of the above-mentioned factors associated with NSSI behaviour, this study further constructed a nomogram prediction model. The model demonstrated good calibration and fit on the training and verification sets, with C-index values of 0.936 and 0.923, average absolute errors between predicted and actual values of 0.092 and 0.105 and Hosmer–Lemeshow test *p* values of 0.452 and 0.523. These results show that the model has high accuracy and reliability in predicting NSSI behaviour in adolescents with depression.

The nomogram prediction model has broad prospects and potential for clinical application. Through this model, medical staff can accurately assess the risk of NSSI behaviour in patients with adolescent depression to formulate personalised treatment programmes and intervention measures. For patients predicted to be at high risk by the model, medical staff can formulate personalised and enhanced psychological intervention programmes in accordance with specific conditions [22]. For example, for patients with childhood trauma, trauma-focused cognitive behavioural therapy can help them deal with negative emotions and cognitive biases caused by past trauma. For patients who are at high risk because of tense parental relationships, family therapy can be provided, the family communication mode can be improved, family conflicts can be alleviated and the family support function can be enhanced. These personalised treatment programmes help meet the psychological needs of patients and promote the recovery of their mental health. On the basis of the prediction results of the model, medical staff can increase the follow-up frequency of high-risk patients and pay close attention to changes in their psychological state [23]. Through regular evaluation and monitoring, medical staff can identify the risk factors that may induce self-injury behaviour in time and take corresponding treatment measures quickly. For example, for patients with negative mood swings or cognitive impairment, the treatment plan can be adjusted in time, and psychological counselling and drug treatment can be strengthened to reduce the risk of self-injury behaviour.

Family environment has an important influence on adolescents’ mental health. Medical staff can communicate and cooperate with patients’ families in depth. help them realise the importance of the family environment to patients. and guide them to improve family functions actively and create a warm, harmonious and supportive family atmosphere. By improving the family support system, patients’ psychological resilience can be enhanced and the possibility of seeking relief through self-injury behaviour can be reduced. The model is also helpful to the rational distribution of medical resources. By focusing professional resources on patients assessed as high risk, it ensures that these patients receive adequate, professional interventions and treatments. This approach not only improves the utilisation efficiency of medical resources, but also ensures the quality of mental health services for the whole group of adolescent patients with depression and achieves the goal of precise medical care and intervention [24].

Although this study has made achievements in constructing and verifying the nomogram prediction model, it still has some limitations. Its samples originated from a single medical centre, and its sample size is limited (n = 136). Geographical representation is insufficient. Although the training and verification sets were divided and verified internally, they may affect the stability of the model. This study did not involve potential factors, such as social support and coping style, nor did it analyse the behaviour type and frequency of NSSI in layers. Given that its cross-sectional design lacks longitudinal data, clarifying causal relationships and dynamic evolution is difficult. Although the inclusion of neuroimaging indicators is valuable, selection bias may exist, and neural markers and dynamic evolution are not discussed. Future research can expand the sample size, conduct multicentre research and adopt a longitudinal design. Moreover, they can incorporate tools, such as the Multidimensional Perceived Social Support Scale and Coping Style Scale, to quantify potential protective factors and record treatment exposure variables. In addition, they can study the behaviour classification of NSSI and explore the gene–environment–nerve interaction mechanism by combining molecular genetics and epigenetics. Furthermore, future work can analyse the interaction between neuroimaging and clinical factors, as well as carry out external verification in wide clinical scenarios, such as different regions and varying levels of medical institutions [25].

## Conclusion

This study identified factors associated with NSSI in adolescent depression—parental tension, long depression duration, co-occurring physical diseases, high depression/anxiety severity, childhood trauma, EEG frontal α power, fMRI DLPFC activation and negative life events—and constructed a nomogram model with good predictive efficacy. By integrating psychological–neural–clinical indicators, the model serves as a visual tool for early NSSI risk assessment. Future directions include multicentre validation with diverse samples, longitudinal studies to explore causal dynamics, incorporation of protective factors (such as social support) and clinical implementation for personalised intervention planning.

## Availability of Data and Materials

The data that support the findings of this study are available from the corresponding author upon reasonable request.
